# Polymorphisms in two DNA repair genes (*XPD* and *XRCC1*) – association with age related cataracts

**Published:** 2011-01-12

**Authors:** G. Padma, M. Mamata, K. Ravi Kumar Reddy, T. Padma

**Affiliations:** 1 Department of Genetics, Osmania University, Tarnaka, Hyderabad, A.P, India; 2 Sarojini Devi Eye Hospital and Institute of Ophthalmology, Hyderabad, A.P, India

## Abstract

**Purpose:**

Age related cataract is the leading cause of blindness in the world today. The association between DNA damage to the lens epithelium and the development of lens opacities has been reported in many studies. Polymorphisms of DNA repair enzymes may affect repair efficiency and thereby lead to the development of age related cataract.

**Methods:**

In this study, we aimed to determine the frequency of polymorphisms in two DNA repair enzyme genes, xeroderma pigmentosum complementation group (*XPD*) codon 312 and X-ray complementing group1 (*XRCC1*) codon 399, in a sample of 208 cataract patients (69 with cortical, 69 with nuclear and 70 with posterior sub capsular) and 151 sex and age matched healthy controls. *XPD* genotype was determined by Amplification Refractory Mutation System (ARMS) while *XRCC1* was genotyped using the PCR-RFLP method.

**Results:**

There was a significant difference between frequencies for *XPD*-312 Asn/Asn genotype in cataract patients (21.6%) and healthy controls (13.2%; p=0.03, OR=1.97, 95% CI=1.06–3.63). Considering the types of cataract, *XPD*-312 Asn/Asn genotype was found to be significantly different in patients with cortical (29%) type in comparison to controls (13.2%; p=0.03, OR=2.39, 95% CI=1.11–5.12). No statistically significant difference was found for the genotypic and allelic distributions of the polymorphism in *XRCC1*. The MDR interaction analysis revealed weak synergism between the markers *XPD*-Asp312Asn and *XRCC1*-Arg399Gln contributing to cataract. It also showed that the AA genotype of *XPD*-Asp312Asn polymorphism when present in combination with the GA genotype of *XRCC1*-Arg399Gln had a fivefold and with AA had a fourfold risk for developing cataract.

**Conclusions:**

The present study suggests that a polymorphism in *XPD* codon 312 may be associated with the development of maturity onset cataract. This is the first report on the association of *XPD* Asp312Asn polymorphism with maturity onset cataract.

## Introduction

Age related cataract is the leading cause of blindness in the world today. It is a multifactorial disease caused by the interactions between the gene and environmental factors. Various risk factors such as diabetes, female gender, sunlight or exposure to UV light, or nutritional deficiencies have been implicated in the development of cataract [1]. Endogenous oxidative damage to proteins, lipids, and DNA has been hypothesized to be important etiologic factors in aging and the development of systemic diseases such as cancer, atherosclerosis, and ocular disorders including cataract, glaucoma, uveitis, and age-related macular degeneration [2,3].

Association between oxidative stress and DNA damage has been well known [4] and many studies have focused on the association between DNA damage to the lens epithelium and the development of lens opacities [5-7]. Ultraviolet (UV) light, one of the contributing factors to cataractogenesis, was shown to cause DNA damage in lens epithelium [8-12]. Chromosomal abnormalities as evidence for such damage have been reported in lens epithelia from patients with cataract [13].

DNA repair enzymes continuously monitor chromosomes to correct damaged nucleotide residues generated by exposure to cytotoxic compounds or carcinogens [14]. Recently, it has been hypothesized in many studies that polymorphisms in DNA repair genes reduce their capacity to repair DNA damage and thereby lead to enhanced cancer or other age-related disease susceptibility [15-19]. Although the exact pathogenetic mechanism of cataract development has not been fully clarified, the involvement of oxidative or UV light damage to DNA and the existence of DNA repair in cataract development indicate the role of DNA repair enzymes.

To date more than 100 DNA repair genes have been identified and their polymorphisms have been reported to be related with some diseases. Among them, polymorphisms of xeroderma pigmentosum complementation group D (*XPD*) and X-ray complementing group I (*XRCC1*) have been studied extensively [16]. *XPD* is required for excision repair of UV damaged DNA and is an important component of nucleotide excision repair (NER) whereas *XRCC1* is involved in single strand breaks and base excision repair (BER). Several studies have pointed out these two genes as good biomarkers of DNA damage [20,21].

We therefore investigated, in the cataract patients the frequency of polymorphisms in *XPD* codon 312 (Asp-Asn) and *XRCC1* coodon 399 (Arg-Gln), which are the most frequent and commonly studied polymorphisms of these two well known DNA repair genes.

## Methods

### Patients and controls

The present case-control study includes a total of 208 patients with age related cataract (69- Cortical cataract [CC], 69- Nuclear cataract [NC], and 70 - Posterior sub capsular cataract [PSC]) and 151 sex and age matched healthy controls. The patients were recruited from the Sarojini Devi Eye Hospital and Institute of Ophthalmology, Hyderabad, India. Diagnosis of different types of cataracts was done based on slit lamp examination by the ophthalmologist following LOC-III classification. The patients and controls were explained about the purpose and outcome of the study and only those who volunteered to participate in the investigations alone were considered. The study was approved by the Institutional ethical committee.

### Inclusion and exclusion criteria

Only patients with primary cataracts were included in the present study. Any patients with a secondary cataract arising due to trauma, action of toxins, inflammations, and degenerative ocular diseases were excluded. In addition, patients with associated conditions like diabetes, hypertension, myopia, glaucoma, thyroid syndromes, and cataract inducing medications (like steroids) were not considered. Controls were selected randomly from the same population and without the history of cataract, diabetes, hypertension, thyroid, and other ocular diseases.

From all the patients and controls included in the study, information pertaining to sex, age, age at onset, duration of disease, type of cataract, information on habits, diet, and a detailed three generation family history was collected using a specific proforma.

### Blood samples and DNA isolation

Venous blood samples were obtained from patients and controls in EDTA vaccutainer tubes. DNA was isolated from these samples using rapid non-enzymatic method described by Lahiri and Nurnberger [22].

### Genotyping of *XPD* Asp312Asn

*XPD* genotype was determined by Amplification Refractory Mutation System (ARMS) where in a 150 bp fragment was generated using reverse primer 5′-CAG GAT CAA AGA GAC AGA CGA GCA GCG C-3′; G allele forward specific primer 5′-GTC GGG GCT CAC CCT GCA GCA CTT CGG C-3′; and A allele forward specific primer 5′-GTC GGG GCT CAC CCT GCA GCA CTT CGA T-3′. An internal control primer was included in each reaction (ARMS A 5′-CCC ACC TTC CCC TCT CTC CAG GCA AAT GGG-3′; ARMS B 5′-GGG CCT CAG TCC CAA CAT GGC TAA GAG GTG-3′) at a 1:5 dilution relative to allele specific primers. Genotypes were typed as GG and AA depending on the development of bands when primers specific for allele G (as GG) or allele A (as AA) were used. Samples were typed as heterozygotes (GA) when bands were seen with both the primers.

### Genotyping of *XRCC1* Arg399Gln

*XRCC1* genotype was determined by PCR RFLP wherein a 615 bp fragment was generated by primer pair forward 5′-TTG TGC TTT CTC TGT GTC CA-3′ and reverse 5′-TCC TCC AGC CTT TTC TGA TA-3′. The PCR reaction was performed in a 10 µl volume of 20 pmoles of each primer, 0.2 mM each dNTP, 1 µl buffer and 0.25 U Taq DNA polymerase, with a initial denaturation of 94 °C for 5 min, followed by 30 cycles of 30 s at 94 °C, 30 s at 61 °C and 45 s at 72 °C and finally 7 min at 72 °C. Following amplification, the PCR products were digested with 2U of Msp1 restriction enzyme and incubated at 37 °C overnight. The wild type Arg allele for codon 399 was determined by the presence of two bands of 374 and 221 bps, while the mutant Gln allele was determined by the presence of the uncut 615 bp band.

### Data analysis

SPSS (version 17) package was used to analyze the data for descriptive statistics and computation of means and standard deviations. χ2 statistics was computed for significance of differences in the distribution of genotypes between patients and controls and also between the cohorts (like sex, obesity, family history, and habits like smoking and alcohol consumption) in the two groups. The frequencies of the marker alleles were estimated by allele counting method and tested for Hardy–Weinberg equilibrium. Odds ratios (OR) and 95% confidence interval (CI) were computed for different combinations of genotypes to estimate the risk contributing to the onset of cataracts.

Multifactor Dimensionality Reduction (MDR) analysis was performed using MDR software to determine the presence of epistatic interaction and the genotypic combination of the two genes that may confer high or low-risk for cataract development. Evaluation of gene-gene and gene-environment interaction was performed using four step process outlined by Moore et al. [23]. The analysis was implemented using open-source MDR software package (v. 1.1.0). Best models with possible combinations of the polymorphisms were considered based on 10-fold cross-validation and maximum testing accuracy.

## Results

Table 1 shows the demographic data of the patients and controls. There was preponderance of female patients (56.3%) as compared to males (43.8%) the frequency being highest in cases of PSC (61.4%) indicating high risk for females for developing age related cataracts specially the posterior sub capsular type. The ages ranged from 35 to 85 years in patients and 40 to 80 years in controls. The mean ages recorded for patients was 58.6±0.40 (CC- 57.8±0.80, NC- 61.45±0.69, PSC-56.1±0.80) and 49.1±0.55 for controls. The mean age at onset of cataract was 57.50±0.39 for the patients in general with delayed onset in patients of NC (60.4±0.68) followed by CC (56.5±0.80) and PSC (54.9±0.81) cases. In 26.9% of patients positive family history of cataracts was reported in contrast to 21.9% reported in controls. The positive family history was reported highest in PSC (34.3%) as compared to other two types (CC-27.5%; NC-18.8%). In general the incidence of smokers was more in cataract patients (28.4%) as compared to controls (23.8%). The frequency of alcoholics was lesser in patients as compared to controls (Table 1).

**Table 1:** Distribution of baseline characteristic features observed in subjects with different types of cataracts and controls.

	**CC**		**NC**		**PSC**		**Total**		**Controls**	
**Cohorts**	**N**	**%**	**N**	**%**	**N**	**%**	**N**	**%**	**N**	**%**
Total	69		69		70		208		151	
Males	29	42.0	35	50.7	27	38.6	91	43.8	94	62.3
Females	40	58.0	34	49.3	43	61.4	117	56.3	57	37.7
Familial	19	27.5	13	18.8	24	34.3	56	26.9	33	21.9
Non-familial	50	72.5	56	81.2	46	65.7	152	73.1	118	78.1
Smokers	19	27.5	19	27.5	21	30.0	59	28.4	36	23.8
Non-smokers	50	72.5	50	72.5	49	70.0	149	71.6	115	76.2
Alcoholoics	11	15.9	15	21.7	11	15.7	37	17.8	40	26.5
Non-alcoholics	58	84.1	54	78.3	59	84.3	171	82.2	111	73.5
Mean age + SEM	57.8± 0.80		61.45± 0.69		56.1± 0.80		58.6± 0.40		49.1± 0.55	
Mean age at onset + SEM	56.5±0.80		60.4± 0.68		54.9±0.81		57.50± 0.39		—	

### *XPD* and *XRCC1* polymorphisms

The genotypic and allelic distributions of the polymorphisms in *XPD* and *XRCC1* in both cases and controls are shown in Table 2.

**Table 2:** Distribution of Polymorphisms in DNA repair genes *XPD* (Asp312Asn) and *XRCC1* (Arg399Gln) and risk estimates for cataract development.

	**Patients**		**Controls**			
** Genotype**	**n**	**%**	**n**	**%**	**O.R (95%CI)**	**p value**
***XPD***						
Asp/Asp	84	40.4	62	41.1	1.66(0.90–3.07)	0.11
Asp/Asn	79	38.0	69	45.7	1.97(1.06–3.63)	0.03*
Asn/Asn	45	21.6	20	13.2	reference	
G (Asp)	0.59		0.64			
A (Asn)	0.41		0.36		0.82 (0.60–1.12)	0.21
**Recessive model**						
Asp/Asp + Asp/Asn	163	78.4	131	86.8		
Asn/Asn	45	21.6	20	13.2	1.81(1.02–3.20)	0.04*
***XRCC1***						
Arg/Arg	90	43.3	75	49.7	1.50(0.81–2.79)	0.20
Arg/Gln	82	39.4	56	37.1	1.23(0.65–2.33)	0.53
Gln/Gln	36	17.3	20	13.2	reference	
G (Arg)	0.63		0.68			
A (Gln)	0.37		0.32		0.80(0.59–1.10)	0.17
**Recessive model**						
Arg/Arg +Arg/Gln	172	82.7	131	86.8		
Gln/Gln	36	17.3	20	13.2	1.37(0.76–2.46)	0.30

The distribution of *XPD*-Asp312Asn genotypes were consistent with Hardy–Weinberg equilibrium among the controls (p=0.995) but not among the patients (p=0.009) indicating possible association of this polymorphism with age related cataracts. It was observed that though the allele frequencies were not different between the patients and controls, there was a significant difference between the frequencies of *XPD*-312 Asn/Asn genotype in cataract patients (21.6%) as compared to controls (13.2%; p=0.03) even after Boneferroni correction (p=0.06). The OR estimates revealed a possible risk of *XPD*-312 Asn/Asn genotype for developing cataract (O.R-1.97, 95% CI-1.06–3.63). Also when the three genotypes were reduced to two by treating the least common genotype as the recessive one, similar results were obtained for the polymorphism (O.R-1.81, 95% CI-1.02–3.20; p=0.04) indicating nearly twofolds risk for Asn/Asn genotype for developing cataracts.

Considering the *XRCC1*-Arg399Gln (G>A) polymorphism, the genotypes were in accordance with Hardy–Weinberg equilibrium among the cases (p=0.083) and controls (p=0.207). No statistically significant difference was found for the genotypic and allelic distributions of the *XRCC1* polymorphism between the patients and controls.

Table 3 shows the distribution of allele and genotype frequencies of *XPD*-Asp312Asn and *XRCC1*-Arg399Gln polymorphisms in controls and cataract types.

**Table 3:** Distribution of genotype and allele frequencies of *XPD* (Asp312Asn) and *XRCC1* (Arg399Gln) polymorphisms in controls and patients with different types of cataract.

** **	**Cataract subtypes**			
**Genotype**	**CC (69)**	**NC (69)**	**PSC (70)**	**Controls (151)**
***XPD***				
Asp/Asp	26–37.7	29–42.0	29–41.4	62–41.1
Asp/Asn	23–33.3	32–46.4	24–34.3	69–45.7
Asn/Asn	20–29.0*	8–11.6	17–24.3	20–13.2
G (Asp)	0.54	0.65	0.59	0.64
A (Asn)	0.46	0.35	0.41	0.36
***XRCC1***				
Arg/Arg	32–46.4	25–36.2	33–47.1	75–49.7
Arg/Gln	26–37.7	31–44.9	25–35.7	56–37.1
Gln/Gln	11–15.9	13–18.8	12–17.1	20–13.2
G (Arg)	0.65	0.59	0.65	0.64
A (Gln)	0.35	0.41	0.35	0.36

*Asp/Asp Vs Asn/Asn significant; OR=2.39, 95% CI=1.11 – 5.12, p=0.03.

Compared with the controls, the allele frequencies of *XPD*-Asp312Asn polymorphism did not differ significantly in the cataract types. However, investigation of the genotype frequencies in different cataract types and controls revealed a significant difference between frequencies for *XPD*-312 Asn/Asn genotype in patients with CC (29%) and controls (13.2%). The OR estimates revealed that *XPD*-312 Asn/Asn genotype had more than twofolds risk for developing CC (OR=2.39, 95% CI=1.11–5.12, p=0.03) even after Boneferroni correction (0.06).

No statistically significant difference was found for the genotypic and allelic distributions of the polymorphism in *XRCC1* gene between controls and cataract types.

### Analysis of epistatic interaction

**Figure 1:**
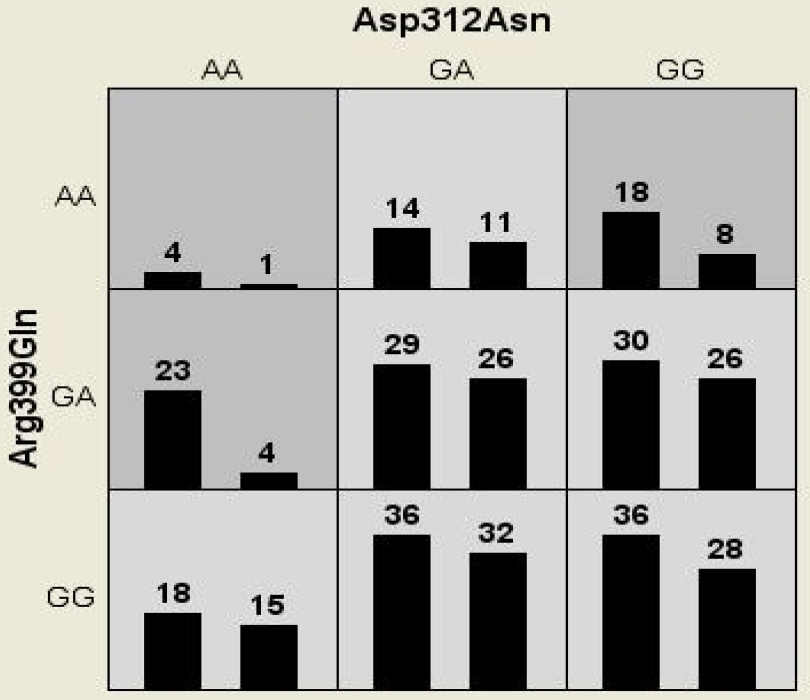
Distribution of high-risk (dark shaded) and low-risk (light shaded) genotypic combinations of the markers studied. The summary of the distribution illustrates the no. of patients (left bars) and controls (right bars) for each genotype combinations.

The present data included polymorphisms Asp312Asn Arg399Gln as the over all best model with maximum testing accuracy of 0.5100 and CV consistency of 10/10. But the combination was not found to be significantly contributing to cataract. High-risk (dark gray) and low-risk (light gray) genotypic combinations were determined based on the threshold value for the present data which was 1.37 (208/151; Figure 1). It was observed that AA genotype of *XPD* Asp312Asn when present in combination with GA genotype of *XRCC1* showed a fivefold risk (23/4) while when present with AA a fourfold risk (4/1) for developing cataract.

**Figure 2:**
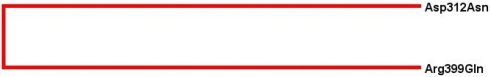
Interaction dendrogram for the two polymorphisms modeled by the MDR method that shows weak synergistic effect of the two polymorphisms on cataract development.

Figure 2 illustrates the MDR interaction information analysis of the two polymorphisms, represented in the form of a dendrogram. The interaction information analysis revealed a weak synergism between the markers *XPD*-Asp312Asn and *XRCC1*-Arg399Gln contributing to cataract.

## Discussion

Successful repair of damaged DNA relies on the coordinated action of many repair enzyme systems. Age dependent decline or imbalance of the activities of the DNA repair enzymes will result in the compromise of the overall capacity of repair for the damaged DNA molecules. This may account for the ever-increasing accumulation of oxidative damage and mutation to DNA in the aging tissues [15,24].

Age-related changes occur also in the human lens. The human lens consists of three metabolically different zones: the epithelium, the cortex and lens nucleus or core [25]. The lens epithelium which covers the anterior surface of the lens is essential for the growth, differentiation, and homeostasis of the lens. In view of the important functions attributed to the lens epithelium, damage to lens epithelial cells has been a focus of many studies to explore cataract development. It is exposed throughout life to a large number of physiologic signals that come through aqueous humor such as hydrogen peroxide and UV light [26,27]. It is suggested that damage to the lens epithelium may result in cataract formation [28,29]. Spector [29] and Spector et al. [5] reported that photochemically induced oxidative stress causes the earliest detectable changes in the epithelial cell redox set point and at the DNA level, and once the epithelial cell layer has been damaged at the DNA and membrane level, cataract formation becomes inevitable. Grabner and Brenner [30] also reported unscheduled DNA repair in human lens epithelium following in vivo and in vitro ultraviolet irradiation.

Common polymorphisms in DNA repair enzymes have been hypothesized to result in reduced capability to repair DNA damage [31,32]. *XRCC1* is a DNA repair gene that is emerging as an essential element in the repair of both damaged bases and SSBs. On the other hand, the *XPD* gene is required for excision repair of UV-damaged DNA and is an important component of NER. Several studies have linked these two polymorphisms with biomarkers of DNA damage [20,21].

To our knowledge, the present study is the first report from Indian population on the association of DNA repair enzymes with age related cataract. We found a possible risk for *XPD* 312 Asn/Asn genotype for developing cataract, especially cortical cataract. But there was no significant association between *XRCC1* polymorphism and cataract development. Interaction analysis also revealed greater risk for AA genotype of *XPD* Asp312Asn polymorphism in combination with AA or GA genotype of *XRCC1* polymorphism (Figure 1).

Previous studies have reported an association between the UV light exposure and the development of cortical cataract. A review of 22 epidemiologic studies by McCarty and Taylor [33], revealed that there is a well documented risk for the development of cortical cataract and UVB exposure [33]. Also, Pendergrass et al. [7] found that in aged mice, most subcapsular and cortical cataracts colocalize with accumulations of nuclei, mitochondria, and DNA and these effects are accompanied at the same sites by the production of reactive oxygen species [7]. Unal et al. [34], suggested that polymorphism in *XPD* codon 751 may be associated with the development of maturity onset cataract. Thus SNPs in *XPD* gene may be expected to have a role in the development of an UV-related disease, like maturity onset cataract. In our study, we found a possible risk of *XPD*-312Asn/Asn genotype in the development of cataract, especially against the development of cortical type cataract. This is the first report on the association of *XPD*- Asp312Asn polymorphism with maturity onset cataract. However, further studies are required to understand the precise mechanisms by which genetic polymorphisms of DNA repair genes influence the process of lens opacification.
